# Aspartate Aminotransferase/Alanine Aminotransferase Ratio: A Predictor of All-Cause Mortality Rate Among Japanese Community-Dwelling Individuals

**DOI:** 10.7759/cureus.52224

**Published:** 2024-01-13

**Authors:** Ryuichi Kawamoto, Asuka Kikuchi, Daisuke Ninomiya, Teru Kumagi

**Affiliations:** 1 Department of Community Medicine, Ehime University Graduate School of Medicine, Toon, JPN

**Keywords:** cohort study, community-dwelling individuals, biomarker, all-cause mortality, ast/alt ratio

## Abstract

Introduction

An elevated ratio of aspartate aminotransferase (AST) to alanine aminotransferase (ALT) not only independently affects aging-related health but also plays a critical role in mortality. However, there is limited predictive data on all-cause mortality, particularly in the context of community-dwelling individuals in Japan. This study examined the association between the AST/ALT ratio and survival prognosis in a cohort study using two follow-up studies based on 19-year and 7-year intervals.

Methods

The study included 1,573 male (63 ± 14 years; range, 20-90 years) and 1,980 female participants (65 ± 12 years; range, 19-89 years). The participants were those involved in a Nomura cohort study conducted in 2002 (first cohort) and 2014 (second cohort) that continued to participate throughout the follow-up periods (follow-up rates were 90.3% and 97.4% for each cohort). A Cox proportional hazards model was adopted to calculate the multivariate-adjusted hazard ratios (HRs) of death from the baseline health check-up to the follow-up periods while controlling for potential confounding factors.

Results

The follow-up survey revealed that there were 473 male deaths (30.1% of total male participants) and 432 female deaths (21.8% of total female participants). The univariate Cox regression analysis showed that HRs for all-cause mortality were greater for participants in higher AST/ALT ratio quartiles (*p* < 0.001). The multivariate Cox regression analysis with adjusted variables showed a significant association between those in the fourth AST/ALT ratio quartile (HR: 1.83, 95% confidence interval, 1.46-2.29) and the risk of all-cause mortality. This association holds irrespective of gender, age, and elevated gamma-glutamyl transpeptidase, particularly in the case of participants with a body mass index < 25 kg/m^2^ without a history of cardiovascular disease or diabetes.

Conclusions

Our results reveal that an elevated AST/ALT ratio is an independent factor that can predict the risk of all-cause mortality among community-dwelling individuals.

## Introduction

Alanine aminotransferase (ALT), aspartate aminotransferase (AST), and gamma-glutamyl transferase (GGT) are major indicators of liver damage [1]. Clinical practices have been testing for these transaminases on a regular basis for decades, but until recently, there has not been enough research done on their potential as mortality predictors. The liver, myocardial tissue, skeletal muscle, brain, liver, kidney, and other tissues, to a lesser degree, are among the tissues that contain AST. On the other hand, ALT is primarily present in the liver and is present in skeletal muscle at much lower concentrations [2]. The significance of the AST/ALT ratio was first described in 1957 [3], and since then, it has often been cited as a marker for various chronic liver diseases, such as alcoholism [4], primary biliary cirrhosis [5], hepatitis C [6], viral liver cirrhosis [7], and autoimmune liver disease [8]. The AST/ALT ratio can also be used to evaluate the degree of hepatic steatosis [9] and insulin resistance [10].

For instance, Maeda et al. [11] suggested that there is a correlation between an elevated AST/ALT ratio and low body mass index (BMI) and malnutrition and that related outcomes tend to be more severe in patients who have suffered acute heart failure. Researchers have also indicated that there is an independent association between elevated AST/ALT ratios and an increased risk of developing cardiovascular diseases (CVD) and all-cause mortality in patients with type 2 diabetes [12], hypertension [13], and heart failure [14,15]. A study based on a general population that participated in a community-based health screening with a 10-year follow-up showed that a high AST/ALT ratio is a new non-traditional risk inflicting CVD episodes [16]. However, there is insufficient knowledge of the predictive abilities of the AST/ALT ratio regarding all-cause mortality among the general population.

Therefore, this study aims to investigate the potential role of the AST/ALT ratio as an independent marker of long-term all-cause mortality among community-dwelling individuals in Japan.

## Materials and methods

Study design and participants

**Figure 1:**
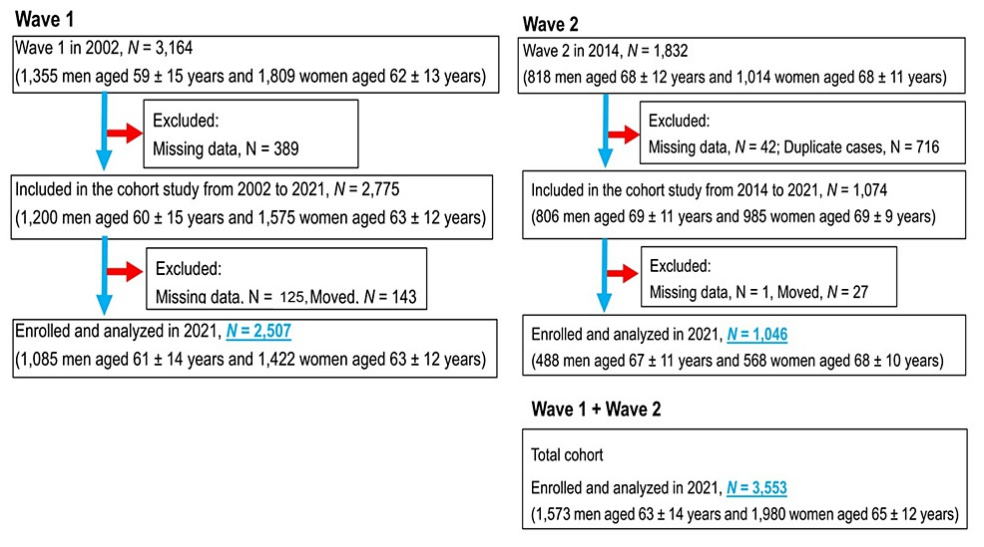
Flowchart showing the process of enrollment and exclusion of participants during previous studies

This longitudinal study involved Japanese participants living in the community of Nomura, Japan, which is primarily rural and has a population of 11,000 inhabitants. The participants underwent annual health check-ups conducted at the community level. The current prospective cohort analysis is a component of the Nomura study [17,18], which was conducted in 2002 (first cohort) and 2014 (second cohort). In Figure 1, we have presented a flowchart that explains participant enrollment and exclusion. Specific inclusion and exclusion criteria were not utilized, but all participants had undergone an annual check-up with approval and responded to questions prepared by medical personnel. This implies that they were not at imminent risk of death, nor did they have severe dementia at baseline. Elderly individuals residing in nursing homes or retirement facilities were excluded from the study. Additionally, inpatients receiving routine treatments at baseline were also excluded. The first and second cohorts comprised 3,164 and 1,832 participants, respectively. All participants were aged between 19 and 90 years. Of the total sample, 2,507 participants in the first cohort and 1,046 in the second cohort underwent baseline physical examinations and follow-ups (follow-up rates: 90.3% and 97.4%). Participants provided information on lifestyle habits, such as smoking, alcohol consumption, and the use of medication for hypertension, diabetes, and dyslipidemia, using a self-administered questionnaire. The questions were formulated by referencing those prepared by the Japanese Ministry of Health, Labor, and Welfare for health check-ups in Japan. A follow-up survey was conducted at a 19-year interval for the first cohort and at a 7-year interval for the second cohort. This study focused on assessment data for the first and second cohorts (n = 3,553). The vital status of participants was verified through Japan's Basic Resident Register, a database containing information about Japanese citizens, to ascertain whether they were alive or deceased. The Nomura studies, however, did not furnish details regarding the causes of death or the emergence of new CVD [17,18].

The Ehime University Hospital's Institutional Review Board (IRB) evaluated and approved this study (approval no. 1903018). Written and informed consent was provided by each participant. This article was previously posted to the medRxiv preprint server on June 7, 2022.

Evaluation of risk factors

First, participants’ baseline weight, height, and other anthropometric indices were measured. BMI was calculated by dividing participants’ weight (kg) by their height (m^2^). Smoking status (pack-years) was estimated as the number of years a person has been a smoker multiplied by the average number of packs smoked per day. Participants were categorized as non-smokers, ex-smokers, light smokers (<20 pack-years), or heavy smokers (>20 pack-years). Daily alcohol consumption was measured on the basis of a unit of sake (22.9 g of ethanol). Accordingly, participants were classified as non-drinkers, occasional drinkers (<1 unit/day), light daily drinkers (1-2 units/day), or heavy daily drinkers (2-3 units/day). No participant reported consuming more than 3 units per day. The systolic blood pressure (SBP) and diastolic blood pressure (DBP) of participants were also measured using an automated sphygmomanometer. They were asked to rest for at least five minutes, following which an appropriately sized cuff was placed on their right upper arm while they remained seated. Statistical analysis was applied to the average of two consecutive readings. Participants were asked to maintain an overnight fast prior to the measurement of their triglyceride (TG), high-density lipoprotein cholesterol (HDL-C), low-density lipoprotein cholesterol (LDL-C), serum uric acid (SUA), blood glucose (BG), GGT, ALT, and AST levels. The detailed process of calculating the AST/ALT ratio was described as follows: (AST (IU/L))/(ALT (IU/L)). To estimate the glomerular filtration ratio (eGFR) of participants, the chronic kidney disease epidemiology collaboration (CKD-EPI) formula was modified with Japanese coefficients. We used the following equation for males with creatinine (Cr) ≤ 0.9 mg/dL: 141 × (Cr/0.9) ^-0.411 ^× 0.993 ^age^ × 0.813; we used the following equation for males with Cr > 0.9 mg/dL: 141 × (Cr/0.9)^ -1.209^ × 0.993 ^age ^× 0.813 We used the following equation for females with Cr ≤ 0.7 mg/dL: 144 × (Cr/0.7) ^-0.329 ^× 0.993 ^age^ × 0.813; we used the following equation for females with Cr > 0.7 mg/dL: 144 × (Cr/0.7) ^-1.209^ × 0.993^ age ^× 0.813 [19].

Participants were considered to have hypertension if they exhibited SBP ≥ 140 mmHg and DBP ≥ 90 mmHg, or if they were on antihypertensive medication. Participants were deemed to have hypertriglyceridemia if their TG levels were ≥ 150 mg/dL, low HDL cholesterolemia if their HDL-C levels were ≤ 40 mg/dL, and hyperlipidemia if their LDL-C levels were ≥ 140 mg/dL or they were on antidyslipidemic medication. Participants were considered diabetic if their BG was ≥ 126 mg/dL and they were on antidiabetic medication. They were categorized as hyperuricemic if their SUA was ≥ 7.0 mg/dL and they consumed SUA-lowering medication. An eGFR of less than 60 mL/min/1.73 m^2^ was considered an indicator of CKD. Elevated GGT levels in participants were defined as 51 IU/L or higher in males and 31 IU/L or higher in females. CVDs that were tested for were ischemic heart disease, ischemic stroke, and peripheral vascular disease.

Statistical analysis

This study used IBM SPSS Statistics for Windows, Version 26 (Released 2019; IBM Corp., Armonk, New York, United States) software for the statistical analyses. Continuous variables with a normal distribution were presented as mean ± standard deviation (SD), and non-normal variables (including TG, BG, GGT, ALT, and AST) were reported as median and interquartile range. Parameters with non-normal distributions were log-transformed. Participants were divided into four quartiles on the basis of their AST/ALT ratios (for male participants, first quartile: < 1.00; second quartile: 1.00-1.06; third quartile: 1.07-1.13; fourth quartile: ≥ 1.14; for female participants, first quartile: < 1.05; second quartile: 1.05-1.10; third quartile: 1.11-1.18; fourth quartile: ≥ 1.19). Chi-squared tests were conducted to compare categorical variables, and ANOVA tests were performed to compare normally distributed continuous variables. A multivariable analysis based on a Cox proportional hazards model was executed using a forced imputation method, in which all baseline confounders were treated as covariates and the main time variable was age. The consistency of the observed association between AST/ALT ratios and all-cause mortality was examined by conducting subgroup analyses. The predictors of the all-cause mortality rate were determined by identifying the areas under the receiver operating characteristic (ROC) curves for each variable. A ROC curve is a plot of sensitivity (true positive) versus 1 - specificity (false positive) for each examined potential marker. An indicator of an effective marker is a ROC curve that has shifted to the left, with areas under the curve close to unity. Diagonals with areas under ROC curves of close to 0.5 denote non-diagnostic markers. Furthermore, a likelihood ratio test was performed to examine the interactions between the AST/ALT ratio quartiles and subgroup variables. Finally, the effect variable was analyzed using an interaction test, wherein all significant confounding variables (except the effect variable) were adjusted. Two-tailed *p* values < 0.05 were defined as statistically significant.

## Results

Baseline characteristics of participants by categories of AST/ALT ratio

The study included 1,980 female participants (age 65 ± 12 years, age 19-89) and 2,573 male participants (age 63 ± 14 years, age 20-90). The follow-up period was 6,391 days (2,694-6,991 days) in the median (interquartile range). About 905 (25.5%) deaths were confirmed by the follow-up, of which 473 were male (30.1% of all males) and 432 female (21.8% of all females). The baseline characteristics of the participants are arranged by AST/ALT ratio categories in Table 1. Individuals with CKD and advanced age were more likely to report higher AST/ALT ratios. Moreover, there was a greater likelihood of reduced prevalence of obesity, hypertriglyceridemia, low HDL cholesterol, high LDL cholesterol, diabetes, hyperuricemia, and elevated GGT in those with higher AST/ALT ratio quartiles. There were no discernible differences between the prevalence of hypertension and smoking habits.

**Table 1:** Baseline characteristics of participants by AST/ALT ratio categories Data are presented as mean ± standard deviation. Data for triglycerides, blood glucose, GGT, AST, and ALT were skewed and were thus presented as median (interquartile range) values and log-transformed for analysis. *p-values were computed using χ2-tests for categorical variables and ANOVA tests for continuous variables. Values that are significant (*p* < 0.05) are bolded. HDL: High-density lipoprotein; LDL: Low-density lipoprotein; eGFR: Estimated glomerular filtration ratio; GGT: Gamma-glutamyl transferase; AST: Aspartate transaminase; ALT: Alanine transaminase

Characteristics	Baseline AST/ALT ratio categories				
	First	Second	Third	Fourth	
Men	<1.00	1.00-1.06	1.07-1.13	≥1.14	
Women	<1.05	1.05-1.10	1.11-1.18	≥1.19	
Total (n = 3,553)	n = 894	n = 878	n = 883	n = 898	*p*-value*
Male gender, n (%)	394 (44.1)	393 (44.8)	386 (43.7)	400 (44.5)	0.971
Age (years)	58 ± 12	63 ± 12	66 ± 11	68 ± 13	<0.001
Obesity, n (%)	431 (48.2)	241 (27.4)	181 (20.5)	106 (11.8)	<0.001
Body mass index (kg/m^2^)	25.1 ± 3.2	23.4 ± 3.1	22.7 ± 2.8	21.9 ± 2.8	<0.001
Smoking habits (non/ex/light/heavy) (%)	66.1/27.6/2.6/3.7	67.0/24.9/2.7/5.4	65.9/25.3/2.4/6.5	65.3/25.2/3.5/6.1	0.244
Alcohol drinking habits (non/occasionally/light/heavy) (%)	46.4/29.8/14.5/9.3	52.5/26.8/12.6/8.1	52.9/23.6/14.0/9.5	54.0/23.1/13.7/9.2	0.029
History of cardiovascular disease, n (%)	54 (6.0)	75 (8.5)	66 (7.5)	88 (9.8)	0.025
Hypertension, n (%)	508 (56.8)	502 (57.2)	502 (56.9)	505 (56.2)	0.983
Systolic blood pressure (mmHg)	138 ± 20	137 ± 21	138 ± 21	137 ± 22	0.990
Diastolic blood pressure (mmHg)	82 ± 11	81 ± 12	80 ± 11	79 ± 11	<0.001
Antihypertensive medication, n (%)	280 (31.3)	281 (32.0)	278 (31.5)	272 (30.3)	0.887
Hypertriglyceridemia, n (%)	271 (30.3)	156 (17.8)	124 (14.0)	93 (10.4)	<0.001
Triglyceride (mg/dL)	110 (81-163)	95 (71-129)	88 (66-123)	83 (64-114)	<0.001
Low HDL-cholesterolemia, n (%)	60 (6.7)	37 (4.2)	32 (3.6)	23 (2.6)	<0.001
HDL cholesterol (mg/dL)	59 ± 15	63 ± 16	65 ± 17	64 ± 16	<0.001
High LDL cholesterolemia, n (%)	340 (38.0)	286 (32.6)	261 (29.6)	236 (26.3)	<0.001
LDL cholesterol (mg/dL)	123 ± 32	118 ± 30	117 ± 30	113 ± 31	<0.001
Lipid-lowering medication, n (%)	103 (11.5)	96 (10.9)	78 (8.8)	70 (7.8)	0.025
Diabetes, n (%)	131 (14.7)	89 (10.1)	60 (6.8)	59 (6.6)	<0.001
Blood glucose (mg/dL)	101 (92-117)	101 (91-115)	101 (91-114)	99 (89-114)	<0.001
Antidiabetic medication, n (%)	116 (13.0)	81 (9.2)	60 (6.8)	59 (6.6)	<0.001
Chronic kidney disease, n (%)	50 (5.6)	84 (9.6)	85 (9.6)	138 (15.4)	<0.001
eGFR (mL/min/1.73 m^2^)	82.6 ± 16.4	78.0 ± 15.8	77.3 ± 15.5	74.0 ± 16.2	<0.001
Hyperuricemia, n (%)	147 (16.4)	124 (14.1)	116 (13.1)	101 (11.2)	0.014
Serum uric acid (mg/dL)	5.4 ± 1.4	5.2 ± 1.4	5.1 ± 1.4	5.0 ± 1.4	<0.001
Serum uric acid-lowering medication, n (%)	38 (4.3)	36 (4.1)	43 (4.9)	37 (4.1)	0.840
Elevated GGT	400 (44.7)	209 (23.8)	128 (14.5)	105 (11.7)	<0.001
GGT (IU/L)	34 (23-63)	25 (18-41)	21 (16-31)	19 (15-28)	<0.001
AST/ALT ratio	0.96 ± 0.05	1.06 ± 0.03	1.13 ± 0.03	1.24 ± 0.09	<0.001
AST(IU/L)	24 (20-30)	23 (20-28)	22 (19-27)	22 (19-26)	<0.001
ALT (IU/L)	27 (21-37)	19 (16-24)	16 (14-19)	13 (10-15)	<0.001

Optimal AST/ALT ratio value to predict all-cause mortality

**Figure 2:**
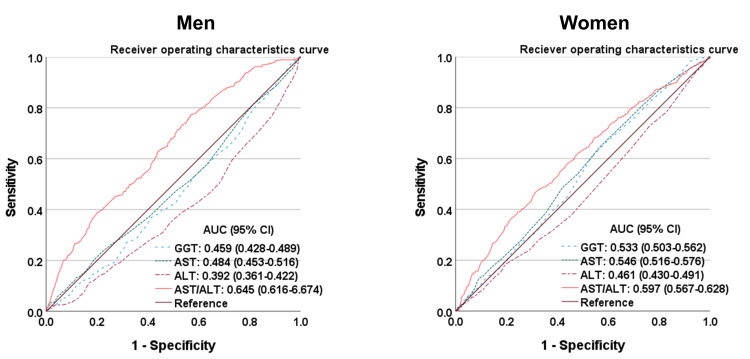
Receiver operating characteristics (ROC) curves that predict all-cause mortality The area under the curve (AUC) for the ratio of aspartate aminotransferase (AST) to alanine aminotransferase (ALT) was significantly greater than the areas for gamma-glutamyl transferase (GGT), AST, and ALT.

Figure 2 shows two ROC curves of liver enzymes (AST, ALT, GGT, and AST/ALT) with the potential to predict all-cause mortality. Notably, the area under the curve of the AST/ALT ratio was greater than that of other liver enzymes.

Kaplan-Meier survival curves of the relationships between AST/ALT ratio quartiles and all-cause mortality by gender

**Figure 3:**
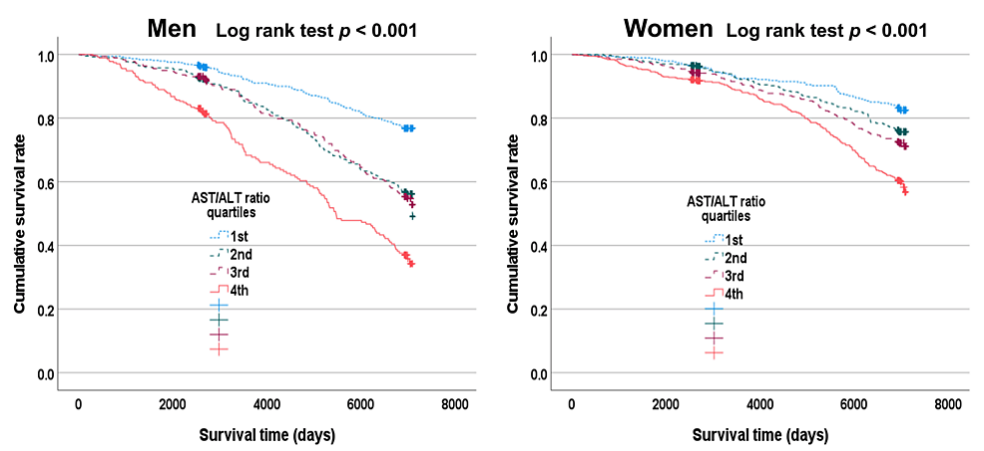
Associations between the aspartate aminotransferase/alanine aminotransferase ratio quartiles and all-cause mortality during the follow-up periods using a survival function *p*-values were obtained using a log-rank test of equality across various strata.

We plotted Kaplan-Meier survival curves for survival days and cumulative survival rates to identify patterns in the relationships between the AST/ALT ratio quartiles and all-cause mortality by gender (Figure 3). The results suggested that the fourth AST/ALT ratio quartile has the lowest cumulative survival rate compared to the other quartiles for both male and female participants (log-rank test: *p* < 0.001).

Hazard ratios and 95% confidence intervals of baseline AST/ALT ratios for all-cause mortality by gender

Table 2 lists the hazard ratios (HRs) and 95% confidence intervals (CIs) for the baseline ALT/AST ratio quartiles for all-cause mortality by gender. The HRs for all-cause mortality were higher for both male and female participants in higher AST/ALT ratio quartiles (*p* < 0.001).

**Table 2:** Hazard ratios and 95% confidence intervals of baseline ALT/AST rate categories for all-cause mortality by gender The multivariate-adjusted HRs were adjusted for gender, age, obesity, alcohol drinking habits, smoking habits, history of cardiovascular disease, hypertension, hypertriglyceridemia, low HDL-cholesterolemia, hyper LDL-cholesterolemia, diabetes, chronic kidney disease, hyperuricemia, and elevated gamma-glutamyl transpeptidase. Values that are significant (*p* < 0.05) are bolded. HR: Hazard ratio; CI: Confidence interval; HDL: High-density lipoprotein; LDL: Low-density lipoprotein

Characteristics (n = 3,553)	n	Event (%)	Non-adjusted HR (95% CI)	*p*-value	Age-adjusted HR (95% CI)	* p*-value	Multivariable-adjusted HR (95% CI)	*p*-value
Men								
ALT/AST ratio quartile								
First quartile	394	61 (15.5)	Reference		Reference		Reference	
Second quartile	395	114 (29.0)	2.37 (1.74-2.24)	<0.001	1.31 (0.95-1.80)	0.096	1.38 (0.99-1.91)	0.057
Third quartile	386	116 (30.1)	2.55 (1.87-3.48)	<0.001	1.06 (0.77-1.46)	0.718	1.24 (0.88-1.73)	0.216
Fourth quartile	400	182 (45.5)	4.53 (3.38-6.05)	<0.001	1.67 (1.23-2.27)	0.001	1.98 (1.42-2.77)	<0.001
*p* for trend	<0.001		<0.001		<0.00		<0.001	
ALT/AST ratio (continuous)	1,573	473 (30.1)	1.56 (1.43-1.69)	<0.001	1.16 (1.05-1.27)	0.002	1.23 (1.11-1.36)	<0.001
Women								
ALT/AST ratio quartile								
First quartile	500	76 8 (15.2)	Reference		Reference		Reference	
Second quartile	485	92 (19.0)	1.39 (1.03-1.88)	0.034	1.04 (0.77-1.42)	0.785	1.15 (0.84-1.58)	0.377
Third quartile	497	106 (21.3)	1.65 (1.23-2.21)	0.001	1.08 (0.80-1.45)	0.627	1.24 (0.90-1.70)	0.187
Fourth quartile	498	158 (31.7)	2.78 (2.11-3.65)	<0.001	1.36 (1.02-1.80)	0.036	1.64 (1.19-2.26)	0.002
*p* for trend	<0.001		<0.001		<0.087		0.010	
ALT/AST ratio (continuous)	1,980	432 (21.8)	1.40 (1.28-1.52)	<0.001	1.14 (1.12-1.15)	<0.001	1.18 (1.06-1.30)	0.001

Next, the analysis was adjusted for gender, age, obesity, alcohol drinking habits, smoking habits, history of CVD, hypertension, hypertriglyceridemia, low HDL-cholesterolemia, hyper-LDL-cholesterolemia, diabetes, CKD, hyperuricemia, and elevated GGT. A significant relationship continues to hold between the fourth AST/ALT ratio quartile and the risk of all-cause mortality in both male (HR: 1.98; 95% CI, 1.42-2.77) and female (HR: 1.64; 95% CI, 1.19-2.26) participants.

Hazard ratios and 95% confidence intervals for baseline AST/ALT ratio quartiles for all-cause mortality as per sub-analyses

Table 3 stratifies participants on the basis of gender, age (<62 years, 62-70 years, ≥71 years, stratified by tertiles of age), BMI (<25 kg/m^2^, ≥25 kg/m^2^), history of CVD, diabetes, elevated GGT, and time till death (<1,095 days, ≥1,095 days). Similar to our previous results, a higher AST/ALT ratio was associated with a greater risk of all-cause mortality, and this held irrespective of gender, age, and elevated GGT and was particularly associated with participants with a BMI < 25 kg/m^2^ and without a history of CVD or diabetes.

**Table 3:** Hazard ratios and 95% confidence intervals of baseline AST/ALT ratio categories for all-cause mortality by sub-analysis The multivariate-adjusted HRs were adjusted for gender, age, obesity, alcohol drinking habits, smoking habits, history of cardiovascular disease, hypertension, hypertriglyceridemia, low HDL-cholesterolemia, hyper LDL-cholesterolemia, diabetes, chronic kidney disease, hyperuricemia, and elevated GGT. Significant values (*p* < 0.05) are presented in bold. HR: Hazard ratio; CI: Confidence interval; GGT: Gamma-glutamyl transpeptidase; HDL: High-density lipoprotein; LDL: Low-density lipoprotein

Characteristics (n = 3,553)	n	Events (%)	Multivariable-adjusted HR (95% CI)	*p*-value	*p* for interaction
Gender					
Male	1,573	473 (30.1)	1.23 (1.11-1.36)	<0.001	0.970
Female	1,980	432 (21.8)	1.18 (1.06-1.30)	0.001	
Age					
19-61 years	1,208	88 (7.3)	1.61 (1.29-2.01)	<0.001	0.398
62-70 years	1,189	245 (20.6)	1.13 (1.00-1.29)	0.043	
71-90 years	1,156	572 (49.5)	1.19 (1.08-1.31)	<0.001	
Body mass index					
<25 kg/m^2^	2,594	671 (25.9)	1.24 (1.14-1.35)	<0.001	0.343
≥25 kg/m^2^	959	234 (24.4)	1.13 (0.99-1.29)	0.094	
History of cardiovascular disease					
No	3,270	750 (22.9)	1.21 (1.12-1.31)	<0.001	0.971
Yes	283	155 (54.8)	1.16 (0.98-1.38)	0.093	
Diabetes					0.414
No	3,214	779 (24.2)	1.23 (1.14-1.33)	<0.001	
Yes	339	126 (37.2)	1.11 (0.92-1.33)	0.275	
Elevated GGT					
No	2,711	700 (25.8)	1.17 (1.08-1.27)	<0.001	0.154
Yes	842	205 (24.3)	1.34 (1.16-1.55)	<0.001	
Time to death					--------
<1,095 days	73	73 (100)	--------	--------	
≥1,095 days	3,480	832 (23.9)	1.20 (1.12-1.29)	<0.001	

## Discussion

This cohort study highlighted the AST/ALT ratio as an independent and significant indicator of all-cause mortality in community-dwelling individuals. After adjusting for confounders, we discovered that the HR (95% CI) of the fourth AST/ALT ratio quartile for all-cause mortality was 1.98 (1.42-2.77) for male participants and 1.64 (1.19-2.26) for female participants. All participants who died within three years of the follow-up period were excluded from the analysis to address the issue of reverse causality. This, however, did not significantly impact the results. There was a significant relationship between the AST/ALT ratio and the all-cause mortality of participants with BMI < 25 kg/m^2^ and without a history of CVD or diabetes, and this held irrespective of gender, age, or elevated GGT. In the era of stratified medicine, this finding has clinical significance because patients whose AST/ALT ratios are 1.15 or higher need to be closely monitored, given their higher risk of mortality. To the best of the authors’ knowledge, few studies have examined the association between AST/ALT ratios and all-cause mortality among Japanese community-dwelling individuals [16].

We found that elevated AST/ALT ratios were significantly associated with an increased risk of all-cause mortality; this result is consistent with several existing studies. However, there is a dearth of reports on this association in the context of community-dwelling individuals. A cohort study on 2,529 type 2 diabetic outpatients found an independent association between the AST/ALT ratio and an increased risk of both all-cause (HR: 1.83; 95% CI, 1.14-2.93) and cardiovascular mortality (HR: 2.60; 95% CI, 1.38-4.90) [12]. A longitudinal cohort study of 3,494 Japanese subjects who underwent community-based health check-ups with a 10-year follow-up indicated that a high AST/ALT ratio (>90%, ≥1.60) represented increased predictability of all-cause mortality (HR: 1.43; 95% CI, 1.04-1.96) and cardiovascular mortality (HR: 2.51; 95% CI, 1.49-4.24) in a general population [16]. More specifically, the study suggested that the AST/ALT ratio was a marker of frailty and a prognostic for all-cause mortality in 1,327 patients who were 65 years and older and hospitalized with heart failure. The association between high AST/ALT ratios (≥1.70) and all-cause mortality continued to hold even after adjusting for confounding factors [14]. Research conducted on 14,220 Chinese hypertensive patients revealed that AST/ALT ratio levels are a potential indicator of all-cause (HR: 1.37, 95% CI, 1.15-1.63) and cardiovascular mortality (HR: 1.32, 95% CI, 1.03-1.68) [13]. Katzke et al. [20] performed a case-cohort study on the prospective European Prospective Investigation into Cancer and Nutrition-Heidelberg cohort (n = 1,632) and reported a significant association between high AST/ALT ratios and risk of prostate cancer (HR: 1.61, 95% CI, 1.10-2.36), all-cause mortality (HR: 1.60, 95% CI, 1.25-2.04), and cancer mortality (HR: 1.67, 95% CI, 1.26-2.23). In sum, the independent association between AST/ALT ratios and all-cause mortality can be observed across various populations, and thus, the AST/ALT ratio could reflect not only liver damage but also systemic damage. The cutoff value of the AST/ALT ratio for all-cause mortality varies among reports, with some reporting a value of 1.0 [12] and others reporting a value of 1.45 [21], 1.6 [13,16], or 1.7 [14]. In our study, there was a significant difference between men and women around the fourth quartile, which was relatively low. Possible reasons for this difference may be due to aging, race, background disease, and differences in measurement kits.

Furthermore, in a retrospective analysis involving 85,658 patients at a Japanese hospital, categorized based on their alcohol consumption, men who were regular drinkers exhibited elevated adjusted HR for all types of cancer in the very high (adjusted HR 1.36; 95% CI 1.13-1.63) and high (adjusted HR 1.26; 95% CI 1.05-1.50) AST/ALT ratio groups [22]. High AST/ALT ratios were linked to unfavorable prognoses in patients with gastric [23], oral and oropharyngeal [24], pancreatic [25], and prostate cancers [26]. Thus, the AST/ALT ratio holds promise as a valuable predictor of survival in cancer patients. Additionally, Qin et al. [27] presented evidence indicating a significant association between AST/ALT ≥ 1.38 and more severe chest CT findings, poorer laboratory results, higher severity of illness scores, and an independent risk factor for the poor prognosis of COVID-19 patients.

However, there is insufficient knowledge about the mechanisms underpinning increased all-cause mortality in individuals with considerably high AST/ALT ratios. Animal-based research conducted on mice has reported declines in oxygen-carrying capacity due to increased AST/ALT ratios and the presence of markers of elevated oxidative stress [28,29]. Therefore, it is reasonable to speculate that a high AST/ALT ratio may increase mortality through oxidative stress. This study highlighted that participants with higher AST/ALT ratios had significantly lower BMIs and were considerably older (*p* < 0.001). This was also observed in participants who were reported as deceased during the follow-up periods. However, in the subgroup analysis, age and BMI had no interaction effect on the relationship between AST/ALT ratios and all-cause mortality. Thus, the AST/ALT ratio can be considered a systemic marker of various conditions that increase the risk of all-cause mortality, possibly through oxidative damage.

The present study offers several advantages. These include its prospective design, the relatively large number of participants involved, the long follow-up period, and the ability to adjust for several important risk factors and potential confounders. The analysis also addressed the possibility of inverse association bias by recruiting individuals who were disease-free at baseline and had not been participants for up to three years. Finally, the results of the sensitivity analysis remained unchanged despite the adjustments.

Limitations of the study

This study is not free from certain limitations. First, we conducted a cross-sectional measurement of the baseline characteristics during the participants’ initial visit. However, AST, ALT, and certain covariates tend to vary during prolonged follow-up periods. Second, our record of deaths is based on the Basic Resident Register, which means that people who moved away from the area during the survey period may have been excluded. Third, the study focused on Japanese participants, and thus, our findings may not be generalizable to other ethnic groups. Fourth, the baseline included many confounding factors that have been reported to be associated with mortality (including medication, underlying diseases, and lifestyle modifications). Finally, we did not collect information on participants’ history of acute or chronic liver disease or the use of associated medications. Therefore, the possibility that liver disease affects AST/ALT ratios cannot be overlooked. Further research on the impact of these unevaluated factors is required.

## Conclusions

Elevated AST/ALT ratios were an independent factor associated with the risk of all-cause mortality among community-dwelling individuals. The precise mechanism underlying this association remains unclear. However, it appears to be unrelated to conventional cardiovascular risk factors, including age, BMI, smoking habits, alcohol consumption, blood pressure, diabetes, and lipid levels. Consequently, these results, combined with the fact that tests for serum AST and ALT are widely and rapidly available, easy to interpret, and inexpensive, suggest that AST/ALT ratios can help identify community residents who are at a high risk of death and therefore in need of early intensive care.
